# *trans*-Di-μ-carbonyl-bis­{carbon­yl[η^5^-2,3,4,5-tetra­methyl-1-(5-methyl-2-fur­yl)cyclo­penta­dien­yl]ruthenium(I)}(*Ru*—*Ru*)

**DOI:** 10.1107/S1600536809026075

**Published:** 2009-07-11

**Authors:** Hong Wang, Gui-Ying Dong, Zhi-Hong Ma, Xiao-Huan Liu, Jin Lin

**Affiliations:** a Hebei Institute of Food Quality, Supervision Inspection and Research, Shijiazhuang 050051, People’s Republic of China; b College of Chemical Engineering and Biotechnology, Hebei Polytechnic University, Tangshan 063009, People’s Republic of China; c College of Basic Medicine, Hebei Medical University, Shijiazhuang 050017, People’s Republic of China; d College of Chemistry & Materials Science, Hebei Normal University, Shijiazhuang 050016, People’s Republic of China

## Abstract

In the crystal structure of the title compound, [Ru_2_(C_14_H_17_O)_2_(CO)_4_], each Ru^I^ atom is connected to one end-on and two bridging carbonyl groups and one cyclo­penta­dienyl ring. The two Ru atoms are connected into binuclear complexes *via* two bridging carbonyl groups, forming four-membered rings which are located on centres of inversion. The Ru—Ru distance of 2.7483 (11) Å corresponds to a single bond. The two carbonyl groups in these binuclear complexes are *trans*-oriented.

## Related literature

For the crystal structures of related ruthenium complexes, see: Schumann *et al.* (2002 ▶); Bailey *et al.* (1978 ▶); Möhring & Coville (2006 ▶); King (1976 ▶); Arndt (2002 ▶).
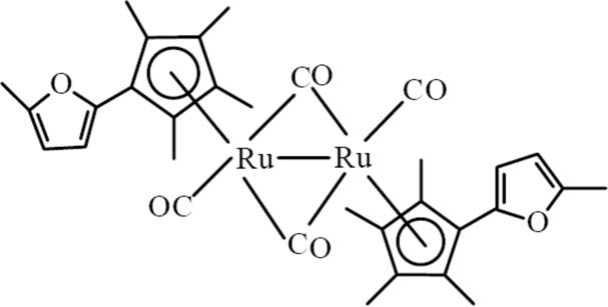


## Experimental

### 

#### Crystal data

[Ru_2_(C_14_H_17_O)_2_(CO)_4_]
*M* *_r_* = 716.73
Monoclinic, 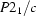

*a* = 8.504 (3) Å
*b* = 16.978 (7) Å
*c* = 10.223 (4) Å
β = 102.220 (6)°
*V* = 1442.5 (10) Å^3^
*Z* = 2
Mo *K*α radiation
μ = 1.09 mm^−1^
*T* = 273 K
0.15 × 0.11 × 0.08 mm

#### Data collection

Bruker SMART CCD area-detector diffractometer
Absorption correction: multi-scan (*SADABS*; Bruker, 1998 ▶) *T*_min_ = 0.854, *T*_max_ = 0.918
7123 measured reflections
2552 independent reflections
2448 reflections with *I* > 2σ(*I*)
*R*_int_ = 0.028

#### Refinement

*R*[*F* ^2^ > 2σ(*F* ^2^)] = 0.036
*wR*(*F* ^2^) = 0.088
*S* = 1.28
2552 reflections
181 parameters
H-atom parameters constrained
Δρ_max_ = 0.72 e Å^−3^
Δρ_min_ = −0.74 e Å^−3^

### 

Data collection: *SMART* (Bruker, 1998 ▶); cell refinement: *SAINT* (Bruker, 1998 ▶); data reduction: *SAINT*; program(s) used to solve structure: *SHELXS97* (Sheldrick, 2008 ▶); program(s) used to refine structure: *SHELXL97* (Sheldrick, 2008 ▶); molecular graphics: *SHELXTL* (Sheldrick, 2008 ▶); software used to prepare material for publication: *SHELXTL*.

## Supplementary Material

Crystal structure: contains datablocks I, global. DOI: 10.1107/S1600536809026075/nc2148sup1.cif

Structure factors: contains datablocks I. DOI: 10.1107/S1600536809026075/nc2148Isup2.hkl

Additional supplementary materials:  crystallographic information; 3D view; checkCIF report

## Figures and Tables

**Fig. 1.**
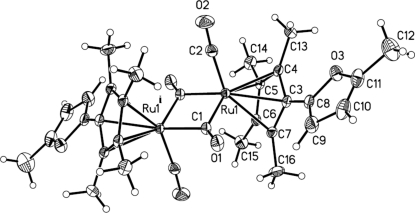
The structure of the title compound with labeling and displacement ellipsoids drawn at the 30% probability level (symmetry code: i=-x+1,-y,-z).

## supplementary crystallographic information

### Comment

Cyclopentadienyl metal complexes have been extensively investigated since ferrocene has been discovered. Replacement of the hydrogen atoms by other substituents alters both the steric and electronic influences of the *µ *^5^-cyclopentadienyl ring, resulting in differing reactivity and stability of the substituted cyclopentadienyl metal complexes. (King, 1976 and Arndt, 2002). Especially for metallocene polymerization catalysts, the steric and electronic effects of cyclopentadienyl ring substituents greatly influence the catalytic activity (Bailey *et al.*, 1978 and Möhring & Coville, 2006).

In the crystal structure of the title compound the Ru atoms is connected to one cyclopentadienyl ring and the carbon atoms of one end-on and two bridging carbonyl groups. The Ru atoms are connected via the bridging carbonyl groups into dimers, which are located on centres of inversion. The Ru1 *Cg*1 distance is 1.9151 Å, where *Cg*1 is the centroid of the cyclopentadiene ring. The Ru—Ru bond distance of 2.7483 (11) Å] agree with that observed in analogous structures (2.7510 (10) Å; Schumann *et al.*, 2002) The two cyclopentadienyl rings are parallel and the two briding carbonyl groups exhibit a *trans* conformation.

### Experimental

A solution of C_5_Me_4_C_5_H_5_O(0.285 g, 1.41 mmol) and Ru_3_(CO)_12_ (0.3 g, 0.47 mmol) in xylene (30 ml) was refluxed for 12 h. The solvent was removed under vacuum and the residue was chromatographed on an Al2O3 column using petroleum ether/CH2Cl2 (1:8) as eluent. The red band was collected and after several days red crystals were obtained (yield 0.189 g, 37.4%). Analysis calculated for Ru_2_C_32_H_34_O_6_: *C* 53.62, H 4.78%; found: C 53.59, H 4.80%.

### Refinement

The H atoms were positioned with idealized geometry [C—H = 0.93 Å (0.96 Å for methy H atoms) and were refined using a riding model with *U*_iso_(H) =1.2*U*_eq_(C)] (1.5 for methyl H atoms).

### Crystal data

**Table:**

[Ru_2_(C_14_H_17_O)_2_(CO)_4_]	*F*(000) = 724
*M**_r_* = 716.73	*D*_x_ = 1.650 Mg m^−^^3^
Monoclinic, *P*2_1_/*c*	Mo *K*α radiation, λ = 0.71073 Å
Hall symbol: -P 2ybc	Cell parameters from 1828 reflections
*a* = 8.504 (3) Å	θ = 5.5–24.8°
*b* = 16.978 (7) Å	µ = 1.09 mm^−^^1^
*c* = 10.223 (4) Å	*T* = 273 K
β = 102.220 (6)°	Prism, red
*V* = 1442.5 (10) Å^3^	0.15 × 0.11 × 0.08 mm
*Z* = 2	

### Data collection

**Table:**

Bruker SMART CCD area-detector diffractometer	2552 independent reflections
Radiation source: fine-focus sealed tube	2448 reflections with *I* > 2σ(*I*)
graphite	*R*_int_ = 0.028
φ and ω scans	θ_max_ = 25.1°, θ_min_ = 2.4°
Absorption correction: multi-scan (*SADABS*; Bruker, 1998)	*h* = −10→9
*T*_min_ = 0.854, *T*_max_ = 0.918	*k* = −19→20
7123 measured reflections	*l* = −9→12

### Refinement

**Table:**

Refinement on *F*^2^	Primary atom site location: structure-invariant direct methods
Least-squares matrix: full	Secondary atom site location: difference Fourier map
*R*[*F*^2^ > 2σ(*F*^2^)] = 0.036	Hydrogen site location: inferred from neighbouring sites
*wR*(*F*^2^) = 0.088	H-atom parameters constrained
*S* = 1.28	*w* = 1/[σ^2^(*F*_o_^2^) + (0.0263*P*)^2^ + 2.646*P*] where *P* = (*F*_o_^2^ + 2*F*_c_^2^)/3
2552 reflections	(Δ/σ)_max_ = 0.001
181 parameters	Δρ_max_ = 0.72 e Å^−^^3^
0 restraints	Δρ_min_ = −0.74 e Å^−^^3^

### Special details

**Table:**

Geometry. All e.s.d.'s (except the e.s.d. in the dihedral angle between two l.s. planes) are estimated using the full covariance matrix. The cell e.s.d.'s are taken into account individually in the estimation of e.s.d.'s in distances, angles and torsion angles; correlations between e.s.d.'s in cell parameters are only used when they are defined by crystal symmetry. An approximate (isotropic) treatment of cell e.s.d.'s is used for estimating e.s.d.'s involving l.s. planes.
Refinement. Refinement of *F*^2^ against ALL reflections. The weighted *R*-factor *wR* and goodness of fit *S* are based on *F*^2^, conventional *R*-factors *R* are based on *F*, with *F* set to zero for negative *F*^2^. The threshold expression of *F*^2^ > σ(*F*^2^) is used only for calculating *R*-factors(gt) *etc*. and is not relevant to the choice of reflections for refinement. *R*-factors based on *F*^2^ are statistically about twice as large as those based on *F*, and *R*- factors based on ALL data will be even larger.

### Fractional atomic coordinates and isotropic or equivalent isotropic displacement parameters (Å^2^)

**Table:**

	*x*	*y*	*z*	*U*_iso_*/*U*_eq_	
Ru1	0.51265 (3)	0.030918 (17)	0.12603 (3)	0.02286 (12)	
O1	0.1922 (3)	0.0424 (2)	−0.0644 (3)	0.0433 (8)	
O2	0.6179 (5)	0.1919 (2)	0.0632 (4)	0.0637 (11)	
O3	0.2618 (4)	0.15332 (18)	0.3848 (3)	0.0422 (7)	
C1	0.3276 (5)	0.0237 (2)	−0.0361 (4)	0.0285 (8)	
C2	0.5750 (5)	0.1294 (3)	0.0811 (4)	0.0353 (9)	
C3	0.3747 (5)	0.0432 (2)	0.2912 (4)	0.0286 (8)	
C4	0.5421 (5)	0.0615 (2)	0.3412 (4)	0.0280 (8)	
C5	0.6327 (5)	−0.0087 (2)	0.3353 (4)	0.0286 (8)	
C6	0.5228 (5)	−0.0684 (2)	0.2787 (4)	0.0288 (8)	
C7	0.3654 (5)	−0.0376 (2)	0.2524 (4)	0.0299 (8)	
C8	0.2398 (5)	0.0956 (2)	0.2882 (4)	0.0330 (9)	
C9	0.0903 (5)	0.1014 (3)	0.2136 (5)	0.0506 (12)	
H9	0.0443	0.0695	0.1417	0.061*	
C10	0.0163 (6)	0.1652 (3)	0.2653 (6)	0.0559 (14)	
H10	−0.0879	0.1832	0.2327	0.067*	
C11	0.1194 (6)	0.1946 (3)	0.3670 (5)	0.0486 (12)	
C12	0.1170 (9)	0.2592 (4)	0.4635 (7)	0.082 (2)	
H12A	0.0222	0.2906	0.4342	0.122*	
H12B	0.2108	0.2915	0.4688	0.122*	
H12C	0.1165	0.2374	0.5501	0.122*	
C13	0.6112 (6)	0.1365 (3)	0.4048 (4)	0.0416 (10)	
H13A	0.5533	0.1803	0.3585	0.062*	
H13B	0.7225	0.1401	0.4000	0.062*	
H13C	0.6021	0.1373	0.4968	0.062*	
C14	0.8093 (5)	−0.0180 (3)	0.3875 (5)	0.0416 (10)	
H14A	0.8298	−0.0277	0.4822	0.062*	
H14B	0.8637	0.0292	0.3705	0.062*	
H14C	0.8480	−0.0617	0.3436	0.062*	
C15	0.5671 (6)	−0.1531 (3)	0.2627 (5)	0.0451 (11)	
H15A	0.5814	−0.1794	0.3475	0.068*	
H15B	0.6654	−0.1555	0.2309	0.068*	
H15C	0.4827	−0.1785	0.1994	0.068*	
C16	0.2141 (6)	−0.0843 (3)	0.2052 (5)	0.0474 (11)	
H16A	0.1693	−0.0718	0.1132	0.071*	
H16B	0.1377	−0.0715	0.2588	0.071*	
H16C	0.2385	−0.1395	0.2132	0.071*	

### Atomic displacement parameters (Å^2^)

**Table:**

	*U*^11^	*U*^22^	*U*^33^	*U*^12^	*U*^13^	*U*^23^
Ru1	0.02253 (18)	0.02590 (19)	0.02035 (18)	−0.00050 (11)	0.00502 (12)	−0.00010 (11)
O1	0.0261 (16)	0.065 (2)	0.0381 (17)	0.0129 (14)	0.0044 (13)	−0.0035 (15)
O2	0.091 (3)	0.0361 (19)	0.060 (2)	−0.0199 (19)	0.007 (2)	0.0109 (16)
O3	0.0409 (17)	0.0434 (17)	0.0429 (18)	0.0105 (14)	0.0103 (14)	−0.0034 (14)
C1	0.031 (2)	0.032 (2)	0.0239 (19)	0.0003 (16)	0.0086 (16)	0.0000 (15)
C2	0.040 (2)	0.037 (2)	0.026 (2)	−0.0032 (18)	0.0023 (17)	0.0005 (17)
C3	0.029 (2)	0.035 (2)	0.0244 (19)	0.0005 (16)	0.0114 (15)	0.0021 (16)
C4	0.0233 (18)	0.039 (2)	0.0216 (19)	−0.0025 (16)	0.0054 (15)	0.0040 (16)
C5	0.030 (2)	0.035 (2)	0.0228 (19)	0.0011 (16)	0.0091 (15)	0.0037 (15)
C6	0.034 (2)	0.031 (2)	0.0224 (19)	0.0012 (16)	0.0085 (16)	0.0035 (15)
C7	0.031 (2)	0.035 (2)	0.0239 (19)	−0.0062 (16)	0.0073 (16)	0.0026 (16)
C8	0.031 (2)	0.042 (2)	0.030 (2)	0.0006 (18)	0.0151 (17)	0.0002 (18)
C9	0.031 (2)	0.076 (4)	0.046 (3)	0.006 (2)	0.008 (2)	−0.009 (2)
C10	0.039 (3)	0.074 (4)	0.059 (3)	0.021 (3)	0.021 (2)	0.013 (3)
C11	0.048 (3)	0.050 (3)	0.053 (3)	0.020 (2)	0.024 (2)	0.009 (2)
C12	0.087 (5)	0.065 (4)	0.097 (5)	0.034 (3)	0.029 (4)	−0.013 (4)
C13	0.046 (3)	0.043 (3)	0.035 (2)	−0.011 (2)	0.0085 (19)	−0.0113 (19)
C14	0.028 (2)	0.058 (3)	0.036 (2)	0.0045 (19)	0.0018 (18)	0.008 (2)
C15	0.060 (3)	0.033 (2)	0.042 (3)	0.006 (2)	0.009 (2)	0.0045 (19)
C16	0.039 (3)	0.048 (3)	0.053 (3)	−0.019 (2)	0.006 (2)	0.001 (2)

### Geometric parameters (Å, °)

**Table:**

Ru1—C2	1.842 (4)	C7—C16	1.502 (6)
Ru1—C1^i^	2.020 (4)	C8—C9	1.341 (6)
Ru1—C1	2.033 (4)	C9—C10	1.410 (7)
Ru1—C4	2.222 (4)	C9—H9	0.9300
Ru1—C3	2.260 (4)	C10—C11	1.310 (8)
Ru1—C5	2.268 (4)	C10—H10	0.9300
Ru1—C6	2.286 (4)	C11—C12	1.477 (8)
Ru1—C7	2.297 (4)	C12—H12A	0.9600
Ru1—Ru1^i^	2.7483 (11)	C12—H12B	0.9600
O1—C1	1.170 (5)	C12—H12C	0.9600
O2—C2	1.149 (5)	C13—H13A	0.9600
O3—C8	1.376 (5)	C13—H13B	0.9600
O3—C11	1.378 (5)	C13—H13C	0.9600
C1—Ru1^i^	2.020 (4)	C14—H14A	0.9600
C3—C7	1.425 (5)	C14—H14B	0.9600
C3—C4	1.441 (5)	C14—H14C	0.9600
C3—C8	1.447 (6)	C15—H15A	0.9600
C4—C5	1.427 (6)	C15—H15B	0.9600
C4—C13	1.492 (6)	C15—H15C	0.9600
C5—C6	1.416 (6)	C16—H16A	0.9600
C5—C14	1.493 (6)	C16—H16B	0.9600
C6—C7	1.409 (6)	C16—H16C	0.9600
C6—C15	1.506 (6)		
			
C2—Ru1—C1^i^	92.69 (17)	C4—C5—Ru1	69.7 (2)
C2—Ru1—C1	93.75 (17)	C14—C5—Ru1	125.9 (3)
C1^i^—Ru1—C1	94.61 (16)	C7—C6—C5	109.3 (3)
C2—Ru1—C4	93.23 (16)	C7—C6—C15	125.7 (4)
C1^i^—Ru1—C4	127.74 (15)	C5—C6—C15	124.7 (4)
C1—Ru1—C4	136.60 (15)	C7—C6—Ru1	72.5 (2)
C2—Ru1—C3	108.97 (17)	C5—C6—Ru1	71.2 (2)
C1^i^—Ru1—C3	152.57 (15)	C15—C6—Ru1	127.2 (3)
C1—Ru1—C3	100.37 (15)	C6—C7—C3	108.0 (3)
C4—Ru1—C3	37.50 (14)	C6—C7—C16	125.5 (4)
C2—Ru1—C5	114.09 (16)	C3—C7—C16	126.2 (4)
C1^i^—Ru1—C5	94.66 (15)	C6—C7—Ru1	71.7 (2)
C1—Ru1—C5	150.13 (15)	C3—C7—Ru1	70.4 (2)
C4—Ru1—C5	37.04 (14)	C16—C7—Ru1	128.2 (3)
C3—Ru1—C5	61.57 (14)	C9—C8—O3	108.9 (4)
C2—Ru1—C6	150.26 (16)	C9—C8—C3	135.1 (4)
C1^i^—Ru1—C6	92.30 (15)	O3—C8—C3	115.9 (4)
C1—Ru1—C6	115.03 (15)	C8—C9—C10	106.7 (5)
C4—Ru1—C6	61.08 (14)	C8—C9—H9	126.6
C3—Ru1—C6	60.55 (14)	C10—C9—H9	126.6
C5—Ru1—C6	36.24 (14)	C11—C10—C9	108.3 (4)
C2—Ru1—C7	145.09 (17)	C11—C10—H10	125.8
C1^i^—Ru1—C7	121.50 (15)	C9—C10—H10	125.8
C1—Ru1—C7	90.47 (15)	C10—C11—O3	109.4 (4)
C4—Ru1—C7	61.45 (14)	C10—C11—C12	135.4 (5)
C3—Ru1—C7	36.42 (14)	O3—C11—C12	115.2 (5)
C5—Ru1—C7	60.63 (14)	C11—C12—H12A	109.5
C6—Ru1—C7	35.80 (14)	C11—C12—H12B	109.5
C2—Ru1—Ru1^i^	94.75 (13)	H12A—C12—H12B	109.5
C1^i^—Ru1—Ru1^i^	47.51 (11)	C11—C12—H12C	109.5
C1—Ru1—Ru1^i^	47.10 (11)	H12A—C12—H12C	109.5
C4—Ru1—Ru1^i^	170.90 (11)	H12B—C12—H12C	109.5
C3—Ru1—Ru1^i^	141.76 (10)	C4—C13—H13A	109.5
C5—Ru1—Ru1^i^	134.53 (10)	C4—C13—H13B	109.5
C6—Ru1—Ru1^i^	110.03 (10)	H13A—C13—H13B	109.5
C7—Ru1—Ru1^i^	112.96 (10)	C4—C13—H13C	109.5
C8—O3—C11	106.6 (4)	H13A—C13—H13C	109.5
O1—C1—Ru1^i^	137.2 (3)	H13B—C13—H13C	109.5
O1—C1—Ru1	137.4 (3)	C5—C14—H14A	109.5
Ru1^i^—C1—Ru1	85.39 (16)	C5—C14—H14B	109.5
O2—C2—Ru1	174.9 (4)	H14A—C14—H14B	109.5
C7—C3—C4	107.4 (3)	C5—C14—H14C	109.5
C7—C3—C8	126.1 (4)	H14A—C14—H14C	109.5
C4—C3—C8	126.4 (4)	H14B—C14—H14C	109.5
C7—C3—Ru1	73.2 (2)	C6—C15—H15A	109.5
C4—C3—Ru1	69.8 (2)	C6—C15—H15B	109.5
C8—C3—Ru1	125.1 (3)	H15A—C15—H15B	109.5
C5—C4—C3	107.8 (3)	C6—C15—H15C	109.5
C5—C4—C13	124.4 (4)	H15A—C15—H15C	109.5
C3—C4—C13	127.4 (4)	H15B—C15—H15C	109.5
C5—C4—Ru1	73.2 (2)	C7—C16—H16A	109.5
C3—C4—Ru1	72.7 (2)	C7—C16—H16B	109.5
C13—C4—Ru1	125.6 (3)	H16A—C16—H16B	109.5
C6—C5—C4	107.4 (3)	C7—C16—H16C	109.5
C6—C5—C14	126.7 (4)	H16A—C16—H16C	109.5
C4—C5—C14	125.8 (4)	H16B—C16—H16C	109.5
C6—C5—Ru1	72.6 (2)		
			
C2—Ru1—C1—O1	−87.0 (5)	C6—Ru1—C5—C4	−116.8 (3)
C1^i^—Ru1—C1—O1	180.0 (6)	C7—Ru1—C5—C4	−80.5 (2)
C4—Ru1—C1—O1	11.7 (6)	Ru1^i^—Ru1—C5—C4	−174.79 (17)
C3—Ru1—C1—O1	23.0 (5)	C2—Ru1—C5—C14	−59.5 (4)
C5—Ru1—C1—O1	72.3 (6)	C1^i^—Ru1—C5—C14	35.5 (4)
C6—Ru1—C1—O1	85.3 (5)	C1—Ru1—C5—C14	143.2 (4)
C7—Ru1—C1—O1	58.3 (5)	C4—Ru1—C5—C14	−120.2 (5)
Ru1^i^—Ru1—C1—O1	180.0 (6)	C3—Ru1—C5—C14	−158.9 (4)
C2—Ru1—C1—Ru1^i^	93.01 (17)	C6—Ru1—C5—C14	123.1 (5)
C1^i^—Ru1—C1—Ru1^i^	0.0	C7—Ru1—C5—C14	159.3 (4)
C4—Ru1—C1—Ru1^i^	−168.28 (16)	Ru1^i^—Ru1—C5—C14	65.0 (4)
C3—Ru1—C1—Ru1^i^	−156.93 (13)	C4—C5—C6—C7	−1.5 (4)
C5—Ru1—C1—Ru1^i^	−107.7 (3)	C14—C5—C6—C7	174.9 (4)
C6—Ru1—C1—Ru1^i^	−94.71 (15)	Ru1—C5—C6—C7	−62.9 (3)
C7—Ru1—C1—Ru1^i^	−121.66 (14)	C4—C5—C6—C15	−175.8 (4)
C1^i^—Ru1—C2—O2	−115 (5)	C14—C5—C6—C15	0.5 (6)
C1—Ru1—C2—O2	150 (5)	Ru1—C5—C6—C15	122.8 (4)
C4—Ru1—C2—O2	13 (5)	C4—C5—C6—Ru1	61.4 (2)
C3—Ru1—C2—O2	48 (5)	C14—C5—C6—Ru1	−122.2 (4)
C5—Ru1—C2—O2	−19 (5)	C2—Ru1—C6—C7	113.5 (4)
C6—Ru1—C2—O2	−16 (5)	C1^i^—Ru1—C6—C7	−147.0 (2)
C7—Ru1—C2—O2	54 (5)	C1—Ru1—C6—C7	−50.8 (3)
Ru1^i^—Ru1—C2—O2	−163 (5)	C4—Ru1—C6—C7	80.4 (2)
C2—Ru1—C3—C7	174.1 (2)	C3—Ru1—C6—C7	37.2 (2)
C1^i^—Ru1—C3—C7	−45.5 (4)	C5—Ru1—C6—C7	118.3 (3)
C1—Ru1—C3—C7	76.5 (2)	Ru1^i^—Ru1—C6—C7	−101.8 (2)
C4—Ru1—C3—C7	−116.4 (3)	C2—Ru1—C6—C5	−4.8 (4)
C5—Ru1—C3—C7	−78.1 (2)	C1^i^—Ru1—C6—C5	94.7 (2)
C6—Ru1—C3—C7	−36.5 (2)	C1—Ru1—C6—C5	−169.1 (2)
Ru1^i^—Ru1—C3—C7	48.8 (3)	C4—Ru1—C6—C5	−37.9 (2)
C2—Ru1—C3—C4	−69.5 (3)	C3—Ru1—C6—C5	−81.1 (2)
C1^i^—Ru1—C3—C4	70.8 (4)	C7—Ru1—C6—C5	−118.3 (3)
C1—Ru1—C3—C4	−167.2 (2)	Ru1^i^—Ru1—C6—C5	139.9 (2)
C5—Ru1—C3—C4	38.2 (2)	C2—Ru1—C6—C15	−124.6 (4)
C6—Ru1—C3—C4	79.8 (2)	C1^i^—Ru1—C6—C15	−25.1 (4)
C7—Ru1—C3—C4	116.4 (3)	C1—Ru1—C6—C15	71.1 (4)
Ru1^i^—Ru1—C3—C4	165.20 (18)	C4—Ru1—C6—C15	−157.7 (4)
C2—Ru1—C3—C8	51.4 (4)	C3—Ru1—C6—C15	159.1 (4)
C1^i^—Ru1—C3—C8	−168.2 (3)	C5—Ru1—C6—C15	−119.8 (5)
C1—Ru1—C3—C8	−46.2 (4)	C7—Ru1—C6—C15	121.9 (5)
C4—Ru1—C3—C8	121.0 (4)	Ru1^i^—Ru1—C6—C15	20.1 (4)
C5—Ru1—C3—C8	159.2 (4)	C5—C6—C7—C3	0.7 (4)
C6—Ru1—C3—C8	−159.2 (4)	C15—C6—C7—C3	175.0 (4)
C7—Ru1—C3—C8	−122.7 (5)	Ru1—C6—C7—C3	−61.4 (3)
Ru1^i^—Ru1—C3—C8	−73.8 (4)	C5—C6—C7—C16	−173.6 (4)
C7—C3—C4—C5	−1.3 (4)	C15—C6—C7—C16	0.7 (7)
C8—C3—C4—C5	175.3 (4)	Ru1—C6—C7—C16	124.3 (4)
Ru1—C3—C4—C5	−65.3 (3)	C5—C6—C7—Ru1	62.1 (3)
C7—C3—C4—C13	−174.0 (4)	C15—C6—C7—Ru1	−123.7 (4)
C8—C3—C4—C13	2.6 (6)	C4—C3—C7—C6	0.4 (4)
Ru1—C3—C4—C13	121.9 (4)	C8—C3—C7—C6	−176.3 (4)
C7—C3—C4—Ru1	64.0 (3)	Ru1—C3—C7—C6	62.2 (3)
C8—C3—C4—Ru1	−119.4 (4)	C4—C3—C7—C16	174.6 (4)
C2—Ru1—C4—C5	−127.1 (3)	C8—C3—C7—C16	−2.0 (7)
C1^i^—Ru1—C4—C5	−31.2 (3)	Ru1—C3—C7—C16	−123.6 (4)
C1—Ru1—C4—C5	133.9 (2)	C4—C3—C7—Ru1	−61.8 (2)
C3—Ru1—C4—C5	115.4 (3)	C8—C3—C7—Ru1	121.6 (4)
C6—Ru1—C4—C5	37.1 (2)	C2—Ru1—C7—C6	−127.4 (3)
C7—Ru1—C4—C5	78.1 (2)	C1^i^—Ru1—C7—C6	39.7 (3)
Ru1^i^—Ru1—C4—C5	24.2 (7)	C1—Ru1—C7—C6	135.4 (2)
C2—Ru1—C4—C3	117.5 (3)	C4—Ru1—C7—C6	−79.2 (2)
C1^i^—Ru1—C4—C3	−146.6 (2)	C3—Ru1—C7—C6	−117.6 (3)
C1—Ru1—C4—C3	18.5 (3)	C5—Ru1—C7—C6	−36.7 (2)
C5—Ru1—C4—C3	−115.4 (3)	Ru1^i^—Ru1—C7—C6	92.8 (2)
C6—Ru1—C4—C3	−78.3 (2)	C2—Ru1—C7—C3	−9.7 (4)
C7—Ru1—C4—C3	−37.3 (2)	C1^i^—Ru1—C7—C3	157.3 (2)
Ru1^i^—Ru1—C4—C3	−91.2 (6)	C1—Ru1—C7—C3	−107.0 (2)
C2—Ru1—C4—C13	−6.5 (4)	C4—Ru1—C7—C3	38.4 (2)
C1^i^—Ru1—C4—C13	89.4 (4)	C5—Ru1—C7—C3	80.9 (2)
C1—Ru1—C4—C13	−105.4 (4)	C6—Ru1—C7—C3	117.6 (3)
C3—Ru1—C4—C13	−124.0 (5)	Ru1^i^—Ru1—C7—C3	−149.6 (2)
C5—Ru1—C4—C13	120.6 (4)	C2—Ru1—C7—C16	111.5 (4)
C6—Ru1—C4—C13	157.7 (4)	C1^i^—Ru1—C7—C16	−81.5 (4)
C7—Ru1—C4—C13	−161.3 (4)	C1—Ru1—C7—C16	14.2 (4)
Ru1^i^—Ru1—C4—C13	144.8 (5)	C4—Ru1—C7—C16	159.6 (4)
C3—C4—C5—C6	1.7 (4)	C3—Ru1—C7—C16	121.2 (5)
C13—C4—C5—C6	174.7 (4)	C5—Ru1—C7—C16	−157.8 (4)
Ru1—C4—C5—C6	−63.2 (3)	C6—Ru1—C7—C16	−121.2 (5)
C3—C4—C5—C14	−174.7 (4)	Ru1^i^—Ru1—C7—C16	−28.4 (4)
C13—C4—C5—C14	−1.7 (6)	C11—O3—C8—C9	0.3 (5)
Ru1—C4—C5—C14	120.3 (4)	C11—O3—C8—C3	−178.2 (4)
C3—C4—C5—Ru1	64.9 (3)	C7—C3—C8—C9	−29.6 (8)
C13—C4—C5—Ru1	−122.0 (4)	C4—C3—C8—C9	154.4 (5)
C2—Ru1—C5—C6	177.4 (2)	Ru1—C3—C8—C9	64.7 (7)
C1^i^—Ru1—C5—C6	−87.5 (2)	C7—C3—C8—O3	148.4 (4)
C1—Ru1—C5—C6	20.2 (4)	C4—C3—C8—O3	−27.6 (6)
C4—Ru1—C5—C6	116.8 (3)	Ru1—C3—C8—O3	−117.3 (3)
C3—Ru1—C5—C6	78.0 (2)	O3—C8—C9—C10	0.1 (5)
C7—Ru1—C5—C6	36.2 (2)	C3—C8—C9—C10	178.2 (5)
Ru1^i^—Ru1—C5—C6	−58.0 (3)	C8—C9—C10—C11	−0.6 (6)
C2—Ru1—C5—C4	60.7 (3)	C9—C10—C11—O3	0.7 (6)
C1^i^—Ru1—C5—C4	155.7 (2)	C9—C10—C11—C12	−178.8 (6)
C1—Ru1—C5—C4	−96.6 (3)	C8—O3—C11—C10	−0.6 (5)
C3—Ru1—C5—C4	−38.7 (2)	C8—O3—C11—C12	179.0 (5)

Symmetry codes: (i) −*x*+1, −*y*, −*z*.
